# Epidemiology of prediabetes and diabetes in Namibia, Africa: A multilevel analysis

**DOI:** 10.1111/1753-0407.12829

**Published:** 2018-08-28

**Authors:** Victor T. Adekanmbi, Olalekan A. Uthman, Sebhat Erqou, Justin B. Echouffo‐Tcheugui, Meera N. Harhay, Michael O. Harhay

**Affiliations:** ^1^ Division of Population Medicine, School of Medicine Cardiff University Cardiff UK; ^2^ Warwick‐Centre for Applied Health Research and Delivery, Division of Health Sciences University of Warwick Medical School Coventry UK; ^3^ International Health Group, Liverpool School of Tropical Medicine Liverpool UK; ^4^ Department of Medicine Brown University Providence Rhode Island USA; ^5^ Department of Medicine, Division of Endocrinology, Diabetes & Hypertension, Brigham and Women's Hospital Harvard Medical School Boston Massachusetts USA; ^6^ Department of Medicine, Division of Nephrology and Hypertension Drexel University College of Medicine Philadelphia Pennsylvania USA; ^7^ Department of Epidemiology and Biostatistics Drexel University Dornsife School of Public Health Philadelphia Pennsylvania USA; ^8^ Department of Biostatistics, Epidemiology and Informatics, Perelman School of Medicine University of Pennsylvania Philadelphia Pennsylvania USA

**Keywords:** community factors, diabetes, multilevel analysis, Namibia, socioeconomic status, 社区因素, 糖尿病, 多层面分析, 纳米比亚, 社会经济状况

## Abstract

**Background:**

Diabetes is a leading cause of progressive morbidity and early mortality worldwide. Little is known about the burden of diabetes and prediabetes in Namibia, a Sub‐Saharan African (SSA) country that is undergoing a demographic transition.

**Methods:**

We estimated the prevalence and correlates of diabetes (defined as fasting [capillary] blood glucose [FBG] ≥126 mg/dL) and prediabetes (defined by World Health Organization [WHO] and American Diabetes Association [ADA] criteria as FBG 110–125 and 100–125 mg/dL, respectively) in a random sample of 3278 participants aged 35–64 years from the 2013 Namibia Demographic and Health Survey.

**Results:**

The prevalence of diabetes was 5.1% (95% confidence interval [CI]: 4.2–6.2), with no evidence of gender differences (*P =* 0.45). The prevalence of prediabetes was 6.8% (95% CI 5.8–8.0) using WHO criteria and 20.1% (95% CI 18.4–21.9) using ADA criteria. Male sex, older age, higher body mass index (BMI), and occupation independently increased the odds of diabetes in Namibia, whereas higher BMI was associated with a higher odds of prediabetes, and residing in a household categorized as “middle wealth index” was associated with a lower odds of prediabetes (adjusted odds ratio 0.71; 95% credible interval 0.46–0.99). There was significant clustering of prediabetes and diabetes at the community level.

**Conclusions:**

One in five adult Namibians has prediabetes based on ADA criteria. Resources should be invested at the community level to promote efforts to prevent the progression of this disease and its complications.

## Abstract 2

**Highlights**

- There is considerable community‐level clustering in dysglycemia, providing logic for considering community‐based prevention strategies.
- There was a positive association of diabetes with higher socioeconomic status.
- There was a strong association of body mass index with prediabetes and diabetes, and our findings suggest the trends are interlinked.

## Abstract (zh)

**摘要:**

**背景**

在全世界范围内糖尿病都是一种进展性疾病并且是导致患者过早死亡的主要原因。纳米比亚是一个位于撒哈拉以南(Sub‐Saharan African,SSA)的非洲国家,  目前正处于人口转变之中,  对这个国家的糖尿病以及糖尿病前期的负担知之甚少。

**方法**

我们在一个随机样本中(来自2013年纳米比亚人口统计与健康调查,  纳入了3278名年龄35‐64岁的参与者)评估了糖尿病(定义为空腹[毛细血管]血糖[FBG] ≥ 126 mg/dL)与糖尿病前期(根据世界卫生组织[WHO]与美国糖尿病协会[ADA]的标准分别将FBG定义为110‐125与100‐125 mg/dL)的患病率及其相关因素。

**结果**

糖尿病的患病率为5.1%(95%置信区间[CI]:4.2‐6.2),  并且没有证据表明其具有性别差异(*P* = 0.45)。使用WHO标准的糖尿病前期患病率为6.8%(95% CI为5.8‐8.0),  使用ADA标准为20.1%(95% CI为18.4‐21.9)。男性性别、老龄、体重指数(BMI)更高以及工作都可以独立增加纳米比亚人患糖尿病的风险,  而BMI更高还可以导致糖尿病前期的风险更高,  若参与者居住在被归类为“中等财富指数”的家庭中,  那么他们发生糖尿病前期的可能性就比较低(校正后的可能性为0.71;95%置信区间为0.46‐0.99)。在社区层面上,  糖尿病前期与糖尿病都具有显著的聚集性。

**结论**

根据ADA标准在每5名纳米比亚成年人中就有1名糖尿病前期患者。为了努力预防这种疾病及其并发症的进展,  我们应当将更多的资源投入到社区这一层面。

## Introduction

Diabetes is a leading cause of progressive morbidity and early mortality worldwide.^1^, ^2^ Physical inactivity, poor diet, and associated weight gain are well‐recognized precursors to incident diabetes among adults. A growing body of epidemiological data has started to find links between these risk factors and diabetes in Sub‐Saharan Africa (SSA), where, until recently, diabetes was thought to be rare.^3^ Although the accumulating evidence suggests that morbidity and mortality due to diabetes in SSA will likely continue to increase in coming years,^4^, ^5^, ^6^, ^7^, ^8^ there is limited nationally representative individual‐level data to examine the social patterning of diabetes within individual countries.^9^, ^10^, ^11^, ^12^

With some exceptions,^13^ data on the burden of diabetes in SSA are rarely based on nationally representative data, relying principally on hospital‐based studies, local surveys, or extrapolation from neighboring countries or subpopulations using statistical models.^14^, ^15^ Thus, existing studies have lacked the generalizability needed to aid in the development of tailored and targeted prevention and treatment programs. Therefore, the aim of the present study was to provide a detailed examination of the prevalence of diabetes and prediabetes using a nationally representative cross‐sectional survey from Namibia, an upper‐middle income country in SSA in an advanced state of economic growth compared with its neighboring region.^16^

## Methods

### Study population and survey design

The 2013 Namibia Demographic Health Survey (NDHS) was designed to provide nationally representative estimates of key population and health indicators for the country overall, as well as for urban and rural areas.^17^ Participating households were selected using a partial update of the 2011 Namibia Population and Housing Census.^17^ Briefly, in the first stage, 554 enumeration areas (EA; the smallest administrative unit in Namibia; 269 in urban and 285 in rural areas) were selected. In the second stage, 20 households were identified in each of the 554 EAs. For the primary survey, 11 080 households were selected (*n* = 5380 urban and *n* = 5700 rural households), with a 92.3% response rate. Only preselected households were surveyed to prevent sampling bias. For all consenting households, an adapted Demographic and Health Survey (DHS) household household questionnaire was used to collect information on household characteristics. Using this questionnaire, eligible males and females were selected to participate in a more detailed “male survey” or “female survey”, which had several components, including the serologic data used in this study. Specifically, anthropometric and biologic data were collected from all eligible males and females, aged 35–64 years, in a subsample of half the surveyed households selected to participate in the male survey component. Anthropometric measurements (weight, height and waist circumference) were made by trained survey staff using standardized methods (i.e. the same methods and equipment in all households selected for this survey).^17^

### Measurement of blood glucose and diabetes definition

After a fasting period of ≥8 h, NDHS participants had a capillary blood sample obtained from their middle or ring finger. If they were not fasting at the time of the interview, an appointment was made for the next morning to collect and test a fasting capillary blood sample. Capillary fasting blood glucose (FBG) was measured using the HemoCue 201+ blood glucose analyzer (HemoCue, Angelholm, Sweden). The analyzer showed blood glucose measurements in millimole per liter.

Two alternative criteria were used to define diabetes and prediabetes. First, for the primary analysis, the World Health Organization (WHO) cut‐off values were used, which define diabetes as FBG ≥126 mg/dL (7.0 mmol/L) and impaired fasting glycemia (prediabetes) as a FBG between 110 and 125 mg/dL (6.1–6.9 mmol/L).^18^ We examined these data two ways: first, we used the raw data values; then, to account for potential underestimation due to the use of capillary glucose, we modified or adjusted the reported DHS values by 1.1%^13^, ^19^ and presented the results using cut‐off values on these adjusted values. The American Diabetes Association (ADA) criteria were used as an alternative to the WHO criteria; the ADA criteria use the same cut‐off values for diabetes, but have a lower threshold for prediabetes, namely FBG 100–125 mg/dL (5.6–6.9 mmol/L).^20^

### Assessment of socioeconomic factors and geographic location

To assess the socioeconomic position (SEP) of participants, we focused on four of the commonly used SEP indicators that could be derived from questionnaire responses: relative household wealth, education level, employment status, and geographic location (urban vs rural residence).^21^, ^22^

The 2013 NDHS provided a derived wealth index, which was created using a three‐step principal component analysis (PCA) of household assets.^23^, ^24^ This standardized metric is estimated in every DHS survey^25^ and is an asset‐based wealth index that conceptualizes wealth (or economic status) as an underlying unobserved dimension that can be estimated using latent variable techniques.^26^, ^27^ As a standardized metric from a country‐specific distribution, households that score low on this index are poor relative to households within the same country, although absolute poverty is not directly estimated by this index.

Self‐reported level of educational attainment was grouped into four categories: no formal education, primary, secondary, and higher education. Employment status of the participants was grouped into three categories: not working, manual labor, or white collar. Finally, we included a binary variable for geographic location as provided by the 2013 NDHS, which categorized each household as being either urban or rural. The DHS defines urban areas as large cities (capital cities and cities with a population over 1 million), small cities (population > 50 000), and towns (other urban areas). Any locations that did not meet any of these three criteria were assumed to be rural.

### Assessment of community‐level factors

We used the term “community” to describe clustering within the same geographical living environment. Communities were based on sharing a common primary sampling unit (PSU) within the DHS data. We considered the following community‐level factors in our analysis: poverty rate, illiteracy rate, and unemployment rate. The poverty rate was defined as the proportion of households living below the poverty level (wealth index <20%; poorest quintile). Illiteracy rate was defined as the proportion of people in the community with no formal education. The unemployment rate was defined as the proportion of people who are unemployed in the communities. For each community‐level factor, the median value was used to categorize the PSU as high, middle, or low for these factors.

### Ethical considerations

This study was based on analysis of existing survey datasets from the archive of the DHS who granted permission for us to use anonymized data. The instruments and conduct of the 2013 NDHS were approved by the Institutional Review Board (IRB) of ICF Macro International (Fairfax, VA, USA). This research is limited to the use of previously collected anonymized data.

### Statistical analysis

For all analyses, all available participants with data were used (i.e. complete case analysis). Descriptive statistics of the NDHS participants were contrasted by diabetes status using χ^2^ tests for categorical variables and Student's *t*‐test for continuous variables. The prevalence of prediabetes or diabetes was estimated for the whole study population and for population subgroups. The age‐adjusted prevalence of prediabetes and diabetes was obtained using logistic regression models. Prevalence estimates accounted for the complex survey design as well as sampling weights.

Four multivariable multilevel logistic regression models were constructed to assess the individual‐ and community‐level factors associated with prediabetes and diabetes in Namibia.^28^ The initial model (Model 1), did not include any independent variables. The purpose of this model was to decompose the amount of variance that existed at each level (i.e. individual and community levels). In the second model (Model 2), a priori‐selected participant characteristics (i.e. age, sex, body mass index [BMI], education, occupation, and family wealth index) were included. In the third model (Model 3), a priori‐selected community‐level variables (i.e. poverty rate, illiteracy rate, unemployment rate, and urban vs rural locality) were included. The last model (Model 4) included all participant and community variables simultaneously. The effect estimates of the participant and community variables (i.e. fixed effects) are presented as adjusted odds ratios (aOR) with corresponding 95% credible intervals (CrIs), derived using Markov Chain Monte Carlo (MCMC) methods. Measures of random effects included intracluster correlation (ICC) and median odds ratio (MOR).^29^, ^30^ The ICC was calculated by the linear threshold according to the formula used by Snijders and Bosker^31^ whereas the MOR is a measure of unexplained cluster heterogeneity.

Descriptive statistics and prevalence rate analyses were derived using Stata statistical software for Windows version 14 (StataCorp, College Station, TX, USA), and multilevel models were built using MLwiN 2.36^32^ on the platform of Stata statistical software for Windows version 14 using the runmlwin routine. Two‐sided *P* < 0.05 was considered significant.

## Results

Analyses involved up to 3278 participants (59% female), with a mean (±SEM) age of 46.9 ± 0.2 years. Of these 3278 participants, 178 (5.4%) had diabetes and 225 (6.9%) had prediabetes (Table 1). Participants with a diabetic‐range FBG were more likely to be older, obese, within the richest wealth index, and to reside in communities with a low illiteracy rate and in urban areas (Table 1).

**Table 1:** Characteristics of the study population, aged ≥35 years, Namibia, 2013

	All (*n =* 3278)	No diabetes (*n =* 3100)	Diabetes (*n =* 178)	*P*‐value
Individual‐level factors				
Age (years)	46.9 ± 0.2	46.7 ±0.2	49.3 ±0.7	<0.001
Sex				0.527
Female	1916 (58.5)	1816 (58.6)	100 (56.2)	
Male	1362 (41.5)	1284 (42.4)	78 (43.8)	
BMI category				<0.001
Underweight	365 (11.2)	352 (11.5)	13 (7.3)	
Normal weight	1502 (46.2)	1450 (47.2)	52 (29.4)	
Overweight	726 (22.4)	677 (22.0)	49 (27.7)	
Obese	656 (20.2)	593 (19.3)	63 (35.6)	
Education attainment				0.066
No education	521 (16.0)	502 (16.3)	19 (10.7)	
Primary	1092 (33.5)	1036 (33.6)	56 (31.7)	
Secondary	1400 (42.9)	1318 (42.7)	82 (46.3)	
Higher	249 (7.6)	229 (7.4)	20 (11.3)	
Wealth index of family				<0.001
Poorest	572 (17.5)	556 (17.9)	16 (9.0)	
Poorer	599 (18.3)	578 (18.7)	21 (11.8)	
Middle	649 (19.8)	624 (20.1)	25 (14.0)	
Richer	762 (23.2)	709 (22.9)	53 (29.8)	
Richest	696 (21.2)	633 (20.4)	63 (35.4)	
Occupation				0.206
Not working	1721 (54.3)	1622 (54.1)	99 (57.9)	
White collar	559 (17.6)	525 (17.5)	34 (19.9)	
Manual laborer	890 (28.1)	852 (28.4)	38 (22.2)	
Community‐level factors				
Poverty rate				<0.001
Low	1837 (56.1)	1714 (55.3)	123 (69.1)	
Middle	368 (11.2)	347 (11.2)	21 (11.8)	
High	1073 (32.7)	1039 (33.5)	34 (19.1)	
Illiteracy rate				0.004
Low	1463 (44.6)	1362 (43.9)	101 (56.8)	
Middle	820 (25.0)	784 (25.3)	36 (20.2)	
High	995 (30.4)	954 (30.8)	41 (23.0)	
Unemployment rate				0.404
Low	1126 (34.4)	1063 (34.3)	63 (35.4)	
Middle	1106 (33.7)	1040 (33.5)	66 (37.1)	
High	1046 (31.9)	997 (32.2)	49 (27.5)	
Place of residence				<0.001
Urban	1530 (46.7)	1422 (45.9)	108 (60.7)	
Rural	1748 (53.3)	1678 (54.1)	70 (39.3)	

Data are given as the mean ± SEM or as *n* (%). Numbers may not sum to the total sample size (*n =* 3278) for certain characteristics because of missing data.

BMI, body mass index.

### Prevalence of prediabetes and diabetes

The age‐adjusted prevalence of prediabetes was 6.7% (95% confidence interval [CI] 5.9–7.9) using WHO criteria and 20.0% (95% CI 18.2–21.8) using ADA criteria. The age‐adjusted prevalence of diabetes was 5.0% (95% CI 4.0–6.0; Tables 2 and 3). The prevalence of dysglycemia (a combination of prediabetes and diabetes) according to WHO and ADA criteria was 13% and 25%, respectively. There were no significant differences in the prevalence of diabetes or prediabetes between male and female participants (Table 2). Participants with a white‐collar job had the highest age‐adjusted prevalence of diabetes compared with those not working and those in the manual job category (Table 2). However, the age‐adjusted prevalence of diabetes among those from the richest families was threefold the prevalence of those from the poorest families (8.5% vs 2.4%; Table 2). The age‐adjusted prevalence of prediabetes was higher among rural than urban dwellers (6.8% vs 6.5%; Table 2).

**Table 2:** Prevalence of prediabetes and diabetes in individuals according to participant characteristics using World Health Organization (WHO) criteria^18^ and modified WHO classification criteria,^13^, ^19^ Namibia, 2013

	Prevalence (%) of prediabetes (95% CI)				Prevalence (%) of diabetes* (95% CI)	
	WHO criteria		Modified WHO criteria		Unadjusted	Age adjusted
	unadjusted	Age adjusted	unadjusted	Age adjusted		
Overall prevalence	6.8 (5.8–8.0)	6.7 (5.9–7.9)	9.2 (8.0–10.4)	9.0 (7.8–10.3)	5.1 (4.2–6.2)	5.0 (4.0–6.0)
Individual‐level factors						
Sex						
Female	7.2 (5.9–8.7)	7.0 (5.6–8.4)	9.4 (8.0–11.1)	9.3 (7.8–10.8)	4.9 (3.8–6.2)	4.7 (3.5–5.9)
Male	6.3 (4.9–8.1)	6.2 (4.6–7.8)	8.7 (7.1–10.7)	8.7 (6.8–10.5)	5.5 (4.1–7.2)	5.4 (3.9–6.9)
BMI category						
Underweight	8.3 (5.5–12.5)	8.2 (4.8–11.6)	12.4 (9.0–16.8)	12.3 (8.4–16.2)	3.4 (1.8–6.3)	3.3 (1.2–5.5)
Normal weight	6.4 (4.9–8.3)	6.3 (4.6–8.0)	8.6 (6.9–10.6)	8.6 (6.7–10.4)	3.1 (2.3–4.2)	3.1 (2.1–4.0)
Overweight	4.3 (3.0–6.2)	4.2 (2.6–5.7)	6.1 (4.4–8.3)	6.0 (4.1–7.9)	6.3 (4.5–8.8)	6.1 (4.1–8.2)
Obese	9.8 (7.3–12.9)	9.5 (6.8–12.2)	12.0 (9.4–15.4)	11.8 (8.9–14.7)	10.0 (7.6–13.2)	9.7 (7.0–12.4)
Education attainment						
No education	7.0 (5.0–9.9)	6.4 (4.0–8.8)	8.7 (6.4–11.9)	8.1 (5.4–10.7)	3.7 (2.1–6.6)	3.1 (1.2–5.0)
Primary	6.8 (5.3–8.7)	6.4 (4.7–8.1)	9.5 (7.8–11.5)	9.0 (7.2–10.9)	4.5 (3.3–6.1)	4.0 (2.7–5.4)
Secondary	5.5 (4.2–7.1)	5.8 (4.3–7.2)	7.9 (6.4–9.8)	8.2 (6.5–10.0)	5.7 (4.4–7.3)	6.0 (4.5–7.5)
Higher	12.4 (8.5–17.7)	12.1 (7.7–16.6)	14.2 (10.1–19.8)	14.1 (9.4–18.8)	6.7 (3.9–11.3)	6.4 (2.9–9.9)
Wealth index of family						
Poorest	6.8 (4.6–10.0)	6.6 (3.9–9.3)	10.9 (8.2–14.3)	10.6 (7.6–13.6)	2.6 (1.5–4.5)	2.4 (1.0–3.9)
Poorer	7.0 (5.0–9.9)	6.9 (4.5–9.4)	9.1 (6.7–12.1)	9.0 (6.3–11.7)	2.8 (1.5–4.9)	2.7 (1.1–4.2)
Middle	5.0 (3.5–7.0)	4.9 (3.2–6.6)	7.8 (6.0–10.1)	7.8 (5.7–9.8)	4.0 (2.5–6.2)	3.9 (2.1–5.7)
Richer	5.6 (4.0–7.8)	5.5 (3.7–7.3)	7.2 (5.4–9.6)	7.1 (5.1–9.2)	7.1 (4.8–10.2)	6.8 (4.3–9.4)
Richest	9.6 (7.3–12.5)	9.5 (7.0–12.1)	11.0 (8.6–13.9)	10.9 (8.3–13.5)	8.6 (6.1–11.9)	8.5 (5.6–11.3)
Occupation						
Not working	7.2 (5.8–8.8)	6.8 (5.2–8.4)	9.9 (8.4–11.7)	9.6 (7.9–11.3)	5.5 (4.2–7.2)	5.1 (3.6–6.7)
White collar	7.8 (5.6–10.9)	8.0 (5.3–10.7)	9.8 (7.4–13.0)	10.0 (7.2–12.9)	5.2 (3.6–7.4)	5.3 (3.4–7.2)
Manual laborer	5.6 (4.1–7.7)	5.8 (4.0–7.6)	7.6 (5.7–10.0)	7.8 (5.7–10.0)	4.0 (2.7–5.8)	4.1 (2.5–5.7)
Community‐level factors						
Poverty rate						
Low	6.8 (5.5–8.3)	6.8 (5.4–8.2)	8.5 (7.1–10.2)	8.6 (7.0–10.1)	6.5 (5.1–8.3)	6.5 (4.9–8.1)
Middle	6.8 (4.5–9.9)	6.3 (3.8–8.9)	10.2 (7.5–13.7)	9.8 (6.8–12.7)	5.5 (2.7–10.6)	5.0 (1.4–8.6)
High	6.9 (5.1–9.4)	6.7 (4.5–8.8)	9.8 (7.7–12.4)	9.6 (7.3–11.9)	2.9 (2.0–4.2)	2.7 (1.6–3.8)
Illiteracy rate						
Low	6.7 (5.3–8.5)	6.7 (5.1–8.3)	9.1 (7.5–10.9)	9.1 (7.4–10.8)	6.1 (4.7–7.9)	6.0 (4.4–7.6)
Middle	6.9 (4.9–9.7)	6.8 (4.4–9.1)	9.4 (7.1–12.3)	9.3 (6.7–11.9)	3.9 (2.6–5.9)	3.8 (2.2–5.4)
High	6.9 (5.2–9.0)	6.6 (4.7–8.4)	9.1 (7.1–11.6)	8.8 (6.7–11.0)	4.6 (2.9–7.2)	4.3 (2.3–6.3)
Unemployment rate						
Low	6.1 (4.6–8.1)	6.3 (4.5–8.1)	7.8 (6.2–9.9)	8.1 (6.2–10.0)	5.1 (3.7–7.0)	5.3 (3.6–7.0)
Middle	7.5 (5.8–9.7)	7.3 (5.4–9.2)	10.0 (8.1–12.4)	9.9 (7.8–12.0)	6.1 (4.4–8.2)	5.8 (4.0–7.6)
High	7.0 (5.2–9.2)	6.6 (4.5–8.7)	9.7 (7.7–12.2)	9.4 (7.1–11.6)	4.3 (2.7–6.5)	3.9 (2.1–5.7)
Place of residence						
Urban	6.4 (5.1–8.1)	6.5 (5.0–8.0)	8.3 (6.8–10.0)	8.4 (6.8–10.0)	6.7 (5.1–8.7)	6.7 (4.9–8.6)
Rural	7.2 (5.7–8.9)	6.8 (5.2–8.5)	9.9 (8.3–11.3)	9.6 (7.8–11.4)	3.7 (2.8–5.0)	3.4 (2.3–4.5)

BMI, body mass index; CI, confidence interval.

* The unadjusted and age‐adjusted prevalence estimates of diabetes are the same using either WHO or modified WHO criteria.

**Table 3:** Prevalence of prediabetes and diabetes in individuals by characteristics using American Diabetes Association classification criteria,^20^ Namibia, 2013

	Prevalence (%) of prediabetes (95% CI)		Prevalence (%) of diabetes (95% CI)	
	Unadjusted	Age‐adjusted	Unadjusted	Age‐adjusted
Overall prevalence	20.1 (18.4–21.9)	20.0 (18.2–21.8)	5.1 (4.2–6.2)	5.0 (4.0–6.0)
Individual‐level factors				
Sex				
Female	21.0 (18.9–23.3)	20.9 (18.7–23.1)	4.9 (3.8–6.2)	4.7 (3.5–5.9)
Male	18.7 (16.3–21.3)	18.7 (16.2–21.2)	5.5 (4.1–7.2)	5.4 (3.9–6.9)
BMI category				
Underweight	19.1 (15.0–24.0)	19.1 (14.6–23.6)	3.4 (1.8–6.3)	3.3 (1.2–5.5)
Normal weight	18.5 (16.1–21.0)	18.5 (16.0–21.0)	3.1 (2.3–4.2)	3.1 (2.1–4.0)
Overweight	17.2 (14.3–20.6)	17.1 (14.0–20.3)	6.3 (4.5–8.8)	6.1 (4.1–8.2)
Obese	27.9 (24.0–32.1)	27.7 (23.6–31.7)	10.0 (7.6–13.2)	9.7 (7.0–12.4)
Education attainment				
No education	22.2 (18.4–26.5)	21.5 (17.5–25.6)	3.7 (2.1–6.6)	3.1 (1.2–5.0)
Primary	19.5 (16.9–22.3)	19.1 (16.4–21.8)	4.5 (3.3–6.1)	4.0 (2.7–5.4)
Secondary	18.8 (16.3–21.6)	19.2 (16.5–21.9)	5.7 (4.4–7.3)	6.0 (4.5–7.5)
Higher	20.1 (18.3–21.9)	24.7 (18.2–31.1)	6.7 (3.9–11.3)	6.4 (2.9–9.9)
Wealth index of family				
Poorest	23.6 (20.2–27.4)	23.4 (19.7–27.1)	2.6 (1.5–4.5)	2.4 (1.0–3.9)
Poorer	18.8 (15.3–22.9)	18.8 (15.0–22.6)	2.8 (1.5–4.9)	2.7 (1.1–4.2)
Middle	17.8 (14.8–21.2)	17.8 (14.6–21.0)	4.0 (2.5–6.2)	3.9 (2.1–5.7)
Richer	17.5 (14.2–21.5)	17.5 (13.9–21.0)	7.1 (4.8–10.2)	6.8 (4.3–9.4)
Richest	22.8 (19.3–26.7)	22.8 (19.1–26.6)	8.6 (6.1–11.9)	8.5 (5.6–11.3)
Occupation				
Not working	20.9 (18.6–23.4)	20.6 (18.2–22.8)	5.5 (4.2–7.2)	5.1 (3.6–6.7)
White collar	21.3 (17.6–25.5)	21.5 (17.6–25.5)	5.2 (3.6–7.4)	5.3 (3.4–7.2)
Manual laborer	18.1 (15.2–21.3)	18.4 (15.4–21.4)	4.0 (2.7–5.8)	4.1 (2.5–5.7)
Community‐level factors				
Poverty rate				
Low	18.7 (16.5–21.1)	18.8 (16.5–21.1)	6.5 (5.1–8.3)	6.5 (4.9–8.1)
Middle	21.3 (16.0–27.8)	20.9 (15.1–26.8)	5.5 (2.7–10.6)	5.0 (1.4–8.6)
High	21.8 (18.9–25.0)	21.6 (18.6–24.7)	2.9 (2.0–4.2)	2.7 (1.6–3.8)
Illiteracy rate				
Low	19.3 (16.9–22.0)	19.4 (16.9–21.9)	6.1 (4.7–7.9)	6.0 (4.4–7.6)
Middle	21.7 (18.0–26.0)	21.7 (17.7–25.7)	3.9 (2.6–5.9)	3.8 (2.2–5.4)
High	19.8 (16.9–23.0)	19.6 (16.6–22.5)	4.6 (2.9–7.2)	4.3 (2.3–6.3)
Unemployment rate				
Low	18.9 (16.0–22.3)	19.2 (16.0–22.4)	5.1 (3.7–7.0)	5.3 (3.6–7.0)
Middle	19.6 (17.1–22.5)	19.5 (16.8–22.2)	6.1 (4.4–8.2)	5.8 (4.0–7.6)
High	21.6 (18.6–25.0)	21.3 (18.1–24.6)	4.3 (2.7–6.5)	3.9 (2.1–5.7)
Place of residence				
Urban	18.2 (15.8–20.7)	18.3 (15.9–20.8)	6.7 (5.1–8.7)	6.7 (4.9–8.6)
Rural	21.8 (19.3–24.4)	21.5 (19.0–24.1)	3.7 (2.8–5.0)	3.4 (2.3–4.5)

BMI, body mass index; CI, confidence interval.

### Correlates of prediabetes

Table 4 lists the individual‐ and community‐level factors associated with prediabetes in multilevel multivariable models. Obese participants were more likely to have prediabetes than those with a normal BMI (aOR 1.82; 95% CrI 1.36–2.37; *P* < 0.001). Moreover, participants from households with a middle wealth index had lower odds of prediabetes than those from the poorest households (aOR 0.71; 95% CrI 0.46–0.99).

**Table 4:** Factors associated with prediabetes in Namibia identified by multilevel multivariable logistic regression models

Variable	Model 1	Model 2	Model 3	Model 4
Fixed effects				
Individual‐level factors				
Age (in years)		1.01 (1.00–1.02)		1.01 (1.00–1.02)
Female (vs male)		1.13 (0.90–1.38)		1.12 (0.90–1.38)
BMI category				
Underweight		0.94 (0.67–1.28)		0.95 (0.67–1.29)
Normal weight		1 (Reference)		1 (Reference)
Overweight		0.98 (0.75–1.25)		0.99 (0.76–1.28)
Obese		1.77 (1.34–2.28)		1.82 (1.36–2.37)
Education attainment				
No education		1 (Reference)		1 (Reference)
Primary		1.08 (0.81–1.43)		1.03 (0.74–1.37)
Secondary		1.11 (0.82–1.49)		1.06 (0.75–1.45)
Higher		1.46 (0.90–2.28)		1.35 (0.79–2.19)
Wealth index of family				
Poorest		1 (Reference)		1 (Reference)
Poorer		0.72 (0.51–0.96)		0.74 (0.53–1.02)
Middle		0.66 (0.46–0.90)		0.71 (0.46–0.99)
Richer		0.62 (0.42–0.86)		0.71 (0.45–1.05)
Richest		0.72 (0.47–1.05)		0.89 (0.53–1.36)
Occupation				
Not working		1 (Reference)		1 (Reference)
White collar		1.24 (0.91–1.64)		1.25 (0.89–1.72)
Manual laborer		1.15 (0.89–1.46)		1.18 (0.88–1.55)
Community‐level factors				
Poverty rate				
Low			1 (Reference)	1 (Reference)
Middle			1.07 (0.71–1.58)	1.15 (0.72–1.73)
High			1.09 (0.77–1.50)	1.04 (0.68–1.50)
Illiteracy rate				
Low			1 (Reference)	1 (Reference)
Middle			1.00 (0.72–1.35)	1.11 (0.81–1.48)
High			0.89 (0.66–1.15)	0.98 (0.71–1.31)
Unemployment rate				
Low			1 (Reference)	1 (Reference)
Middle			0.89 (0.66–1.18)	0.94 (0.68–1.24)
High			0.93 (0.66–1.25)	1.04 (0.70–1.44)
Rural (vs urban)			1.23 (0.89–1.64)	1.27 (0.91–1.72)
Random effects				
Community‐level				
Variance (SE)	0.417 (0.217–0.653)	0.464 (0.253–0.712)	0.446 (0.253–0.664)	0.464 (0.237–0.713)
Intracommunity correlation (%)	11.3	12.4	11.9	12.4
MOR	1.85	1.91	1.89	1.91
Model fit statistics				
Bayesian DIC	3228.51	3059.80	3233.70	3065.06

Data show adjusted odds ratios with 95% credible intervals in parentheses.

Model 1 is the empty model, a baseline model without any independent variable.

Model 2 is adjusted for individual‐level factors.

Model 3 is adjusted for community‐level factors.

Model 4 is adjusted for individual‐ and community‐level factors.

BMI, body mass index; DIC, deviance information criterion; MOR, median odds ratio.

As indicated in Table 4, relative to the empty model, there was significant variation in the odds of having prediabetes (τ = 0.417; 95% CI 0.217–0.653) across communities in Namibia. The ICC indicated that 11.3% of the variance in the odds of prediabetes could be attributed to community‐level factors. These variations across the communities remained statistically significant after controlling for individual‐level factors (in Model 2), community‐level factors (in Model 3) or both (in Model 4). Results of the MOR showed evidence of community‐dependent phenomenon modifying the odds of prediabetes. The MOR for prediabetes was 1.85 in the empty model; this relatively moderate MOR suggests that the clustering effect was moderate. The unexplained community heterogeneity in prediabetes remained relatively unchanged after adding individual‐ and community‐level factors in the final model.

### Correlates of diabetes

Table 5 shows results of multilevel models for individual‐ and community‐level factors associated with diabetes. Among the individual‐level factors, age, sex, BMI, and occupation were significantly associated with the odds of diabetes in the multilevel multivariable model that included all the factors. The odds of diabetes increased by 1.03‐fold (95% CrI 1.01–1.05) for every 1‐year increase in a participant's age. Female participants had lower odds of diabetes than male participants (aOR 0.61; 95% CrI 0.41–0.86). Overweight and obese participants were 76% and 168% more likely to have diabetes, respectively, than those with normal BMI. Participants in the manual job category had lower odds of having diabetes than those not working (aOR 0.62; 95% CrI 0.36–0.99).

**Table 5:** Factors associated with diabetes mellitus in Namibia identified by multilevel multivariable logistic regression models

Variable	Model 1	Model 2	Model 3	Model 4
Fixed effects				
Individual‐level factors				
Age (in years)		1.03 (1.01–1.05)		1.03 (1.01–1.05)
Female (vs male)		0.63 (0.43–0.90)		0.61 (0.41–0.86)
BMI category				
Underweight		1.06 (0.50–0.97)		1.01 (0.46–1.88)
Normal weight		1 (Reference)		1 (Reference)
Overweight		1.79 (1.09–2.73)		1.76 (1.03–2.74)
Obese		2.71 (1.61–4.30)		2.68 (1.58–4.27)
Education attainment				
No education		1 (Reference)		1 (Reference)
Primary		1.30 (0.67–2.44)		1.26 (0.64–2.31)
Secondary		1.30 (0.65–2.50)		1.24 (0.62–2.42)
Higher		1.21 (0.42–2.82)		1.23 (0.43–2.85)
Wealth index of family				
Poorest		1 (Reference)		1 (Reference)
Poorer		1.28 (0.58–2.43)		1.18 (0.50–2.33)
Middle		1.41 (0.65–2.68)		1.24 (0.53–2.57)
Richer		2.28 (1.05–4.30)		2.06 (0.84–4.54)
Richest		3.09 (1.34–6.04)		2.84 (1.02–6.75)
Occupation				
Not working		1 (Reference)		1 (Reference)
White collar		0.67 (0.38–1.09)		0.72 (0.40–1.20)
Manual laborer		0.57 (0.35–0.87)		0.62 (0.36–0.99)
Community‐level factors				
Poverty rate				
Low			1 (Reference)	1 (Reference)
Middle			0.95 (0.41–1.87)	1.31 (0.52–2.71)
High			0.58 (0.28–1.05)	0.96 (0.42–1.91)
Illiteracy rate				
Low			1 (Reference)	1 (Reference)
Middle			0.71 (0.38–1.19)	0.87 (0.45–1.50)
High			0.77 (0.44–1.24)	1.03 (0.54–1.75)
Unemployment rate				
Low			1 (Reference)	1 (Reference)
Middle			1.52 (0.90–2.37)	1.58 (0.80–2.81)
High			1.64 (0.86–2.85)	1.69 (0.69–3.19)
Rural (vs urban)			0.65 (0.34–1.10)	0.73 (0.37–1.29)
Random effects				
Community‐level				
Variance (SE)	1.435 (0.795–2.170)	1.447 (0.764–2.394)	1.501 (0.808–2.311)	1.646 (0.857–2.669)
Intracommunity correlation (%)	30.4	30.5	31.3	33.3
MOR	3.12	3.13	3.20	3.38
Model fit statistics				
Bayesian DIC	1297.02	1198.97	1290.44	1200.39

Data show adjusted odds ratios with 95% credible intervals in parentheses.

Model 1 is the empty model, a baseline model without any independent variable.

Model 2 is adjusted for individual‐level factors.

Model 3 is adjusted for community‐level factors.

Model 4 is adjusted for individual‐ and community‐level factors.

BMI, body mass index; DIC, deviance information criterion; MOR, median odds ratio.

Table 5 shows random effect results from the multilevel analysis of factors associated with diabetes. In Model 1, there was no significant variation in the log odds of diabetes (τ = 1.435; 95% CI 0.795–2.170) in all the communities included in the study. According to the ICC indicated by the calculated intercept variance, 30.4% of the variation could be linked to community‐level factors. In each of the models adjusted for (individual‐level, community‐level and both simultaneously in the final model), the variance across the communities remained statistically significant. The MOR of 3.12 in Model 1, which increased to 3.38 in the final model (Model 4), indicates that the clustering effect is high.

## Discussion

Herein we examined a large population‐based sample of the 2013 NDHS to describe the epidemiology of diabetes and prediabetes in Namibia. To our knowledge, this study provides the first nationally representative estimate of dysglycemia among Namibians that accounts for individual‐ and community‐level factors. We found a relatively low prevalence of diabetes (5%) in Namibia, but a wide discrepancy in prediabetes prevalence depending on the definition used (7% by WHO criteria and 20% by ADA criteria). Of note, the ADA adopted its prediabetes criteria because, compared with the higher WHO cut‐off, a lower threshold for FBG generated prevalence estimates for prediabetes that more closely corresponded to estimates derived from glucose tolerance testing.^20^ In our analysis, the prevalence of diabetes and prediabetes was highest among overweight and obese individuals, with individuals from the highest family wealth index having the highest prevalence of diabetes.

Further, the vast majority of prior studies on dysglycemia among African populations have focused primarily on diabetes, with limited inclusion of prediabetes.^13^, ^33^, ^34^ Our finding of the potentially large burden of prediabetes in Namibia portends a potentially large future epidemic of diabetes, underscoring the need for Namibian health authorities to prepare to manage commonly concurrent burdens of vascular disease and kidney disease among their citizens. Moreover, Namibia and other SSA countries are undergoing a demographic transition that may hasten the population's progression to diabetes; as death from infection declines, these populations age and develop other risk factors, including obesity, that can hasten the onset of diabetes and its complications.^35^ Our findings of significant clustering of diabetes and prediabetes at the community level supports preventative efforts that address communities in addition to individuals, and future studies are needed to determine additional community‐level factors that contribute to dysglycemia risk.

The associations we found between diabetes and age and BMI are similar to those observed around the world. The positive associations we found between these two factors and diabetes have also been observed previously in South Africa,^36^ Nigeria,^37^ and Zambia.^38^

Our findings of an increased odds of diabetes among individuals with the highest family wealth aligns with the epidemiological transition theory, which postulates that the burden of new diseases related to lifestyle would be first concentrated among the wealthy, before shifting to those of a lower SEP. Similar findings have been noted recently in previous studies conducted in some SSA countries.^39^, ^40^, ^41^ One commonly posited explanation for this association is that higher socioeconomic status increases access to high‐calorie foods and decreases the need for physical activity. Future studies are warranted to examine more specific factors that may explain these associations.

There are limitations to this work that must be considered when evaluating the results. First, testing of blood glucose levels was not repeated among survey participants. In the absence of a confirmatory fasting sample, there is a potential for measurement error. Second, because the 2013 NDHS did not conduct a 2‐h oral glucose tolerance test (OGTT) or measure HbA1c among its participants, we relied solely on FBG to classify prediabetes and diabetes. A large‐scale multicountry study conducted by the NCD Risk Factor Collaboration group^42^ indicates that diabetes prevalence based on FBG alone is lower than that based on the combination of FBG, HbA1c and 2‐h OGTT. Other studies^43^, ^44^, ^45^, ^46^, ^47^ have also shown that HbA1c is more sensitive and less susceptible to fluctuations due to stress, acute illness, and diurnal variations than FBG and reflects glucose homeostasis at a given point in time. Therefore, because prevalence estimates of dysglycemia may be higher when using HbA1c and 2‐h OGTT thresholds compared with FBG thresholds,^48^ our results may represent a conservative estimate of the prevalence of diabetes and prediabetes in the population studied. Third, the measures obtained were of capillary blood glucose, which produces disparate estimates of glucose concentration compared with venous blood. However, capillary blood glucose measurement may be the most practical approach in large‐scale studies, especially in resource‐limited areas, and has been used in past studies as large as the Indian Council of Medical Research‐India Diabetes study^49^ and WHO studies.^18^ Fourth, this work was performed in a cross‐sectional sample. Consequently, causal pathways cannot be assumed; rather, we can only describe associations between a priori and conceptually selected variables. Finally, we used an updated release of the NDHS dataset for this analysis (“NMPR61FL”), which included 184 more individuals than were reported in the published NDHS report.^17^

This study has several strengths. First, the DHS program is a well‐standardized and long‐standing program that has rigorously collected nationally representative data in low‐ and middle‐resource settings for decades. Accordingly, our estimate of diabetes in Namibia is consistent with a prior global epidemiologic analysis of diabetes prevalence rates that included the majority of SSA countries.^4^ Second, the sampling framework used in the NDHS follows closely from national census and thus provides a diverse sample from Namibia. Third, there are advantages to studying factors associated with diabetes using a multilevel approach, because community‐level analyses are better equipped to describe the economic and social context in which individuals live and experience health outcomes. This additional level of granularity is needed to facilitate targeted interventions and preventative measures that will be needed to stem the burden of diabetes and other vascular disease in the developing world.

### Conclusions

To summarize, this work adds to a growing evidence base that several countries in SSA are experiencing a rapidly evolving epidemiological transition marked by an increase in chronic diseases.^1^, ^2^ Our results underscore the importance of future public health policies in SSA that shift focus from the management of acute to chronic conditions. Further, our finding of the potentially large burden of prediabetes in Namibia points to the need to develop preventive care and education efforts,^50^ ideally targeting both at‐risk individuals and communities.

## Disclosure

None of the authors has any conflicts of interest to declare.
